# Identification of Exertional Hypoxia and Its Implications in SARS-CoV-2 Pneumonia

**DOI:** 10.4269/ajtmh.20-1012

**Published:** 2020-08-25

**Authors:** Tanweer Hussain, Harman Talat Saman, Zohaib Yousaf

**Affiliations:** Department of Medicine; Hamad Medical Corporation; Doha, Qatar; Acute Medicine; Derbyshire Hospitals Foundation Trust; Derby, England; E-mail: thussain@hamad.qa; Department of Medicine; Hamad Medical Corporation; Doha, Qatar; Barts Cancer Institute; Queen Mary University of London; London, United Kingdom; E-mail: harman.t.saman@gmail.com; Department of Medicine; Hamad Medical Corporation; Doha, Qatar; Dresden International University; Dresden, Germany; E-mail: zohaib.yousaf@gmail.com

Dear Sir,

We read with great interest the recently published article entitled *Feasibility, Reproducibility, and Clinical Validity of a Quantitative Chest X-Ray Assessment for COVID-19*. In this perspective, Orsi et al.^1^ write about the importance of using cheap and readily available tools like the chest X-ray in the evaluation and management of COVID-19 pneumonia. One of the facts highlighted by the authors is that the use of a widely available tool that has rapid execution at the patient’s bedside may have a significantly broader impact on patient management than more sophisticated tools. Resource-limited settings may benefit even more from the use of such tools. We concur with the authors about the use and ensuing impact of readily available, inexpensive, objective bedside tools, for example, transcutaneous monitoring of oxygen saturation (SPO_2_), especially in COVID-19 patients.

Hypoxia is the hallmark of SARS-CoV-2 pneumonia.^2^ The spotlight of COVID-19–associated hypoxia has been on the resting hypoxia associated with the acute phase of illness. As clinical experience in managing SARS-CoV-2 pneumonia increases, there is growing anecdotal evidence that a small but important group of patients with significant lung damage suffers from hypoxia at the point of hospital discharge. A generally accepted discharge criterion is a transcutaneous SPO_2_ of 94% or more in patients who have been off oxygen for 48 hours or more. However, there is variation in deciding discharge criteria concerning oxygen use. In our clinical experience, some of the patients who fulfilled the aforementioned criteria with no dyspnea at rest still complained of breathlessness on minimal exertion.

Exertional hypoxia (EH) is defined as a drop in SPO_2_ to ≤ 88% on a 6-minute walk test.^3^ Exertional hypoxia is associated with a reduced quality of life and is a marker for poor prognosis in interstitial lung disease and chronic obstructive airway disease. It may progress to chronic hypoxemic respiratory failure.^4–6^ Dyspnea associated with EH is a predictor of reduced exercise capacity.^7^ The mechanism of dyspnea caused by EH is not fully understood. An increase in pulmonary vascular resistance and altered ventilatory and circulatory mechanics may play roles.^8^ There is a dearth of literature on the long-term impact of EH. Thus far, there are no national or international guidelines dictating assessment for the presence of EH and the need for ambulatory or long-term home oxygen in patients who recover from SARS-CoV-2 pneumonia.^9–11^

The long-term complications of EH caused by SARS-CoV-2 pneumonia are not yet evident. Long-term EH is likely to be linked to musculoskeletal deconditioning and/or pulmonary hypertension. It is essential to investigate EH to exclude treatable complications such as pulmonary embolism or pulmonary hypertension. In case no underlying reversible cause of EH is identified, intervention, such as ambulatory oxygen and pulmonary rehabilitation (PR), may be important. Pulmonary rehabilitation is an established treatment modality for hypoxia at rest and on exertion in chronic cardiorespiratory diseases.^12^ It focuses on breaking the cycle of progressive exercise limitations secondary to EH and ensuing deconditioning. Therefore, detecting EH at the point of discharge would allow the opportunity to select patients who might benefit from PR and treatment with ambulatory and long-term home oxygen. Consequently, we are of the view that patients who recover from SARS-CoV-2 pneumonia should be assessed for EH at the point of discharge.

## References

[1] Orsi M Oliva G Toluian T Valenti Pittino C Panzeri M Cellina M, 2020 Feasibility, reproducibility, and clinical validity of a quantitative chest X-ray assessment for COVID-19. Am J Trop Med Hyg 103: 822–827. 32618262 10.4269/ajtmh.20-0535 PMC7410416

[2] Chen N 2020 Epidemiological and clinical characteristics of 99 cases of 2019 novel coronavirus pneumonia in Wuhan, China: a descriptive study. Lancet 395: 507–513. 32007143 10.1016/S0140-6736(20)30211-7 PMC7135076

[3] Stolz D 2014 Exertional hypoxemia in stable COPD is common and predicted by circulating proadrenomedullin. Chest 146: 328–338. 24722847 10.1378/chest.13-1967

[4] Visca D 2018 Effect of ambulatory oxygen on quality of life for patients with fibrotic lung disease (AmbOx): a prospective, open-label, mixed-method, crossover randomised controlled trial. Lancet Respir Med 6: 759–770. 30170904 10.1016/S2213-2600(18)30289-3

[5] Mesquita CB Knaut C Caram LMO Ferrari R Bazan SGZ Godoy I Tanni SE, 2018 Impact of adherence to long-term oxygen therapy on patients with COPD and exertional hypoxemia followed for one year. J Bras Pneumol 44: 390–397. 30517340 10.1590/S1806-37562017000000019 PMC6467586

[6] García-Talavera I Figueira-Gonçalves J Gurbani N Pérez-Méndez L Pedrero-García A, 2018 Clinical characteristics of COPD patients with early-onset desaturation in the 6-minute walk test. Pulmonology 24: 275–279. 29910123 10.1016/j.pulmoe.2018.04.007

[7] LoRusso TJ Belman MJ Elashoff JD Koerner SK, 1993 Prediction of maximal exercise capacity in obstructive and restrictive pulmonary disease. Chest 104: 1748–1754. 8252956 10.1378/chest.104.6.1748

[8] Marin JM Montes de Oca M Rassulo J Celli BR, 1999 Ventilatory drive at rest and perception of exertional dyspnea in severe COPD. Chest 115: 1293–1300. 10334142 10.1378/chest.115.5.1293

[9] UPHS, 2020 Criteria for Discharging Patients from Inpatient Care. Available at: http://www.uphs.upenn.edu/cep/COVID/Inpatient%20discharge%20criteria%20update%20415.pdf. Accessed August 9, 2020.

[10] Acep.org, 2020 Discharge: Expected Recovery. Available at: https://www.acep.org/corona/covid-19-field-guide/treatment/discharge-expected-recovery/. Accessed August 9, 2020.

[11] England.nhs.uk, 2020 Reference Guide for Emergency Medicine. Available at: https://www.england.nhs.uk/coronavirus/wp-content/uploads/sites/52/2020/03/C0261-specialty-guide-emergency-medicine-v5-22-April.pdf. Accessed August 9, 2020.

[12] Spruit MA 2013 An official American Thoracic Society/European Respiratory Society statement: key concepts and advances in pulmonary rehabilitation. Am J Respir Crit Care Med 188: e13–e64. 24127811 10.1164/rccm.201309-1634ST

